# Toxoplasmosis chorioretinitis mimicking acute retinal necrosis associated with local corticosteroid

**DOI:** 10.1186/s40942-020-00225-0

**Published:** 2020-06-03

**Authors:** Jason N. Crosson, Sanjana Kuthyar, Jessica G. Shantha, Matthew R. Debiec, Philip W. Laird, Cindy S. Hwang, Hans E. Grossniklaus, Steven Yeh

**Affiliations:** Emory Eye Center, Emory University School of Medicine, 1365 Clifton Rd, Atlanta, GA 30322 USA

**Keywords:** Toxoplasmosis, Acute retinal necrosis, Corticosteroids

## Abstract

**Background:**

The cases discussed highlight the atypical presentation and diagnostic dilemmas of toxoplasmosis with fulminant retinal necrosis and the potentially devastating visual outcomes of toxoplasma chorioretinitis following local corticosteroid exposure.

**Case presentation:**

We report a series of three patients who presented with toxoplasmosis mimicking severe acute retinal necrosis. Patients were between 59 and 77 years old and had been exposed to local corticosteroids preceding our evaluation. All patients demonstrated diffuse retinal whitening with severe vision loss on presentation. Polymerase chain reaction testing (PCR) was diagnostic in two patients, and histopathologic examination of a vitrectomy specimen was diagnostic in one patient. All cases of retinitis resolved with anti-parasitic medication; however, visual acuity failed to improve in all patients due to disease severity and presentation.

**Conclusions:**

Local corticosteroid injection may trigger or exacerbate toxoplasmosis chorioretinitis, leading to fulminant retinal necrosis and severe vision loss. Toxoplasma chorioretinitis should be considered in the differential diagnosis of patients presenting with clinical features of acute retinal necrosis, particularly following local corticosteroid injection regardless of their baseline systemic immune status. Diagnostic vitrectomy may be helpful in patients in whom PCR testing is negative and ocular toxoplasmosis is suspected.

## Introduction

Ocular toxoplasmosis is a common cause of infectious uveitis that most commonly presents with unilateral retinitis adjacent to a chorioretinal scar; however, systemically immunosuppressed individuals may present with atypical lesions consisting of large areas of retinal necrosis without adjacent retinal scarring [1, 2]. Patients who have received local or systemic corticosteroid without concomitant anti-parasitic therapy have also been reported to develop severe retinal necrosis from toxoplasmosis [3]. We describe three patients with fulminant necrotizing retinitis with features mimicking acute retinal necrosis following corticosteroid administration and were subsequently diagnosed with toxoplasmosis. These cases highlight the atypical presentation, diagnostic difficulties, and severe visual morbidity associated with diffuse toxoplasmosis chorioretinitis, which may occur in patients without a prior documented history of systemic or ocular toxoplasmosis.

## Results: report of cases

### Case 1

**Fig. 1:**
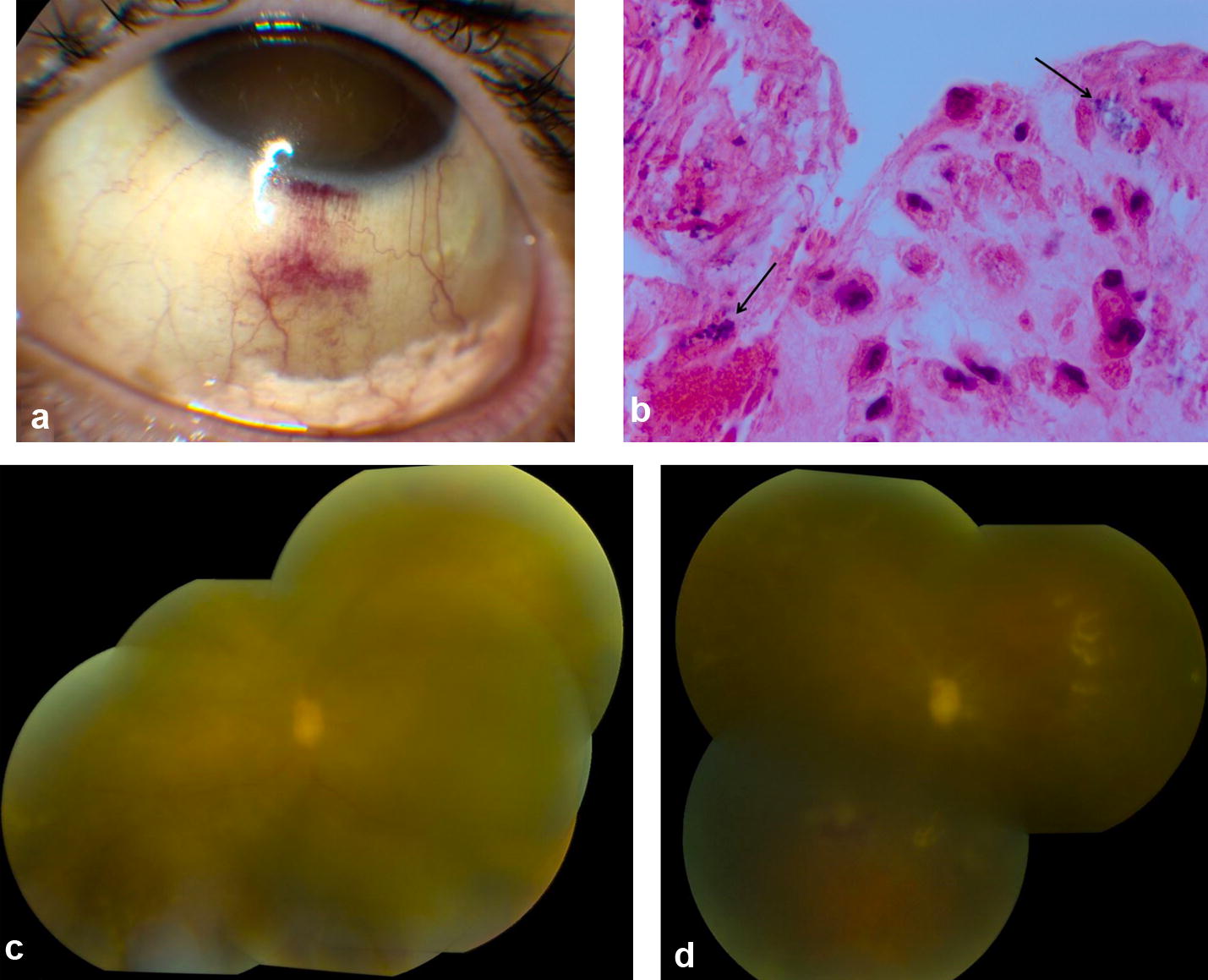
Slit lamp photograph of patient one shows the sub-Tenons triamcinolone acetonide (Kenalog) prior to the development of severe chorioretinitis (**a**). Histopathologic analysis showing toxoplasmosis bradyzoites at 250 × magnification (**b**, arrows). Color fundus photo montage of patient one shows a hazy view secondary to vitritis, disc edema, and patchy retinal whitening (**c**). While the vitreous inflammation and retinal whitening has improved, optic nerve pallor and retinal vascular attenuation are observed (**d**)

**Fig. 2:**
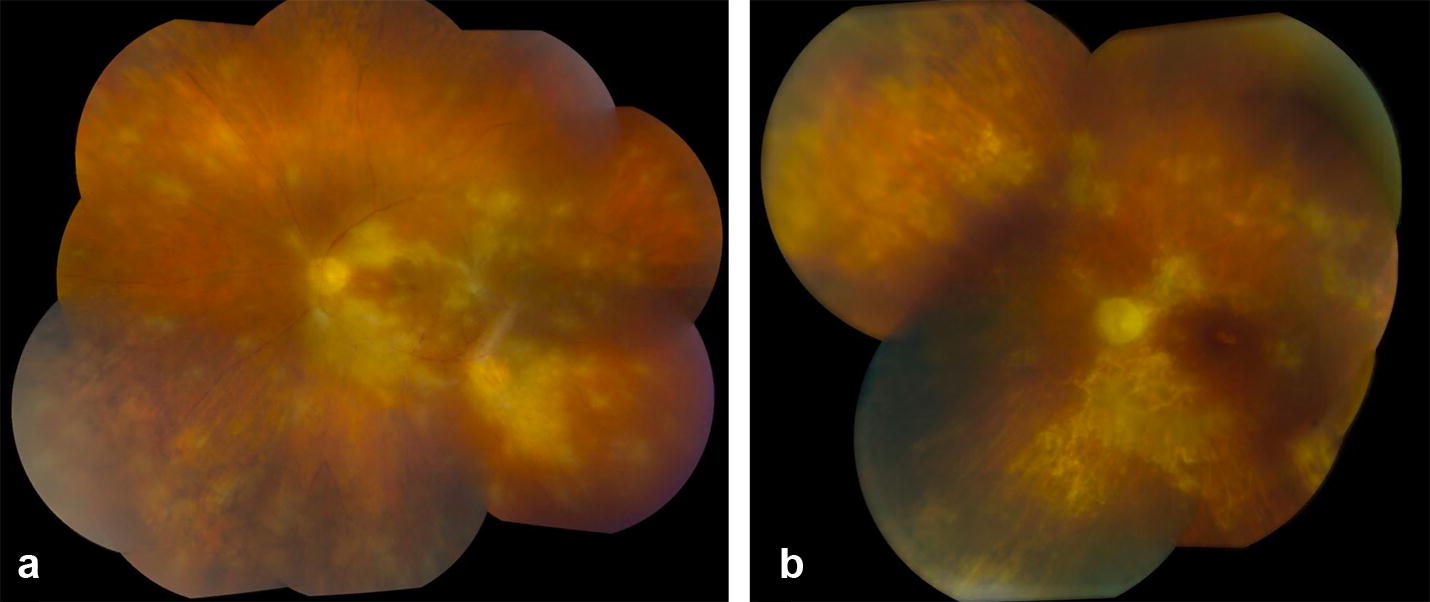
Color fundus photo montage of the left eye of patient two shows diffuse retinal whitening involving the posterior pole and severe vascular attenuation (**a**). With oral and intravitreal anti-parasitic medication, the majority of the retinitis has improved but optic nerve pallor, diffuse retinal pigment epithelial atrophy and severe vascular attenuation are seen (**b**)

A 59-year-old female with advanced glaucoma underwent a bleb revision with bleb needling, mitomycin C, and subtenons triamcinolone for a failed trabeculectomy in the left eye. Three days after her bleb revision, she experienced floaters, pain, and acute loss of vision. She had no prior history of ocular or systemic toxoplasmosis. She was started on oral prednisone and was referred for an evaluation 1 week later when her vision failed to improve. Visual acuities were 20/25 in the right eye and hand motions in the left eye. A relative afferent pupillary defect of the left eye was observed. Slit lamp exam demonstrated a triamcinolone depot in the inferior fornix (Fig. 1) and 2+ anterior chamber cell and vitreous cell in the left eye. Dilated examination of the left eye demonstrated 2+ vitreous haze and patchy retinal whitening temporally in association with sclerotic-appearing vessels within the inferotemporal quadrant. Examination of the right eye was unremarkable. An anterior chamber paracentesis was performed and polymerase chain reaction (PCR) testing for herpes simplex virus (HSV), varicella zoster virus (VZV), cytomegalovirus (CMV) and toxoplasmosis was negative. Toxoplasmosis IgG, toxoplasmosis IgM, and syphilis IgG were also negative. Ceftazidime (2.25 mg/0.1 cc), vancomycin (1 mg/0.1 cc), and foscarnet (2.4 mg/0.1 cc) were injected intravitreally and the patient was started on trimethoprim/sulfamethoxazole (800 mg/160 mg). Five days later, a combined tractional and rhegmatogenous retinal detachment developed, prompting pars plana vitrectomy, endolaser, silicone oil instillation, injection of vancomycin, ceftazidime, voriconazole and foscarnet, and excision of subtenons triamcinolone acetonide. Vitreous specimens were obtained for bacterial and fungal cultures, PCR testing, and cytology. Histopathologic analysis showed bradyzoites consistent with toxoplasmosis (Fig. 1). Therapy with trimethoprim/sulfamethoxazole was continued. The toxoplasmosis chorioretinitis improved, but the visual acuity remained hand motions at the final six-month follow-up.

### Case 2

A 77-year-old male with diabetes mellitus and no prior history of ocular or systemic toxoplasmosis underwent pars plana vitrectomy, membrane peel and intravitreal triamcinolone for an epiretinal membrane in the left eye at an outside institution. He reported significant improvement in his distortion symptoms immediately following surgery. However, one month after surgery, the patient complained of floaters and severe vision loss and was referred to the Emory Eye Center for an evaluation. Presenting visual acuities were 20/20 in the right eye and counting fingers in the left eye. Slit lamp exam revealed 2+ anterior chamber cell and trace anterior vitreous cell in the left eye. Funduscopic exam demonstrated retinitis involving the posterior pole of the left eye (Fig. 2). Examination of the right eye was unremarkable. Due to an initial concern for herpetic acute retinal necrosis, the patient was started oral valacyclovir 1 g three times daily. A vitreous tap for gram stain and culture, as well as PCR testing for HSV, VZV, CMV and toxoplasmosis was performed. Intravitreal foscarnet (2.4 mg/0.1 cc), vancomycin (1 mg/0.1 cc), and ceftazidime (2.25 mg/0.1 cc) were administered. Toxoplasmosis PCR was positive while PCR testing for VZV, HSV, and CMV was negative. Serologic testing showed toxoplasmosis IgG positive at > 250 IU/mL (Normal reference: 0–6.4 IU/mL) and Toxoplasmosis IgM normal at < 0.90 titer. An FTA-ABS was negative. Because of prior sulfa allergy precluding trimethoprim/sulfamethoxazole or sulfadiazine therapy, the patient was treated with a combination of oral azithromycin (500 mg initially, then 250 mg/day) and clindamycin (300 mg four times daily). He also received six intravitreal injections of clindamycin (1 mg/0.1 cc) for a total of six doses over the ensuing month. The retinitis improved, but visual acuity was unimproved with hand motions at the patient’s final 6-month follow-up.

### Case 3

A 74 year-old female with dermatomyositis managed with azathioprine and intravenous immunoglobulin was treated by her ophthalmologist with topical prednisolone acetate 1% for iritis of the right eye. She later developed panuveitis with retinal whitening of the right eye. Because of findings consistent with viral retinitis, the patient was started on valacyclovir, 1 g three times daily and oral prednisone (60 mg). A subtenons triamcinolone injection (40 mg/1 cc) was administered. She was subsequently referred to our institution when her vision continued to deteriorate.

**Fig. 3:**
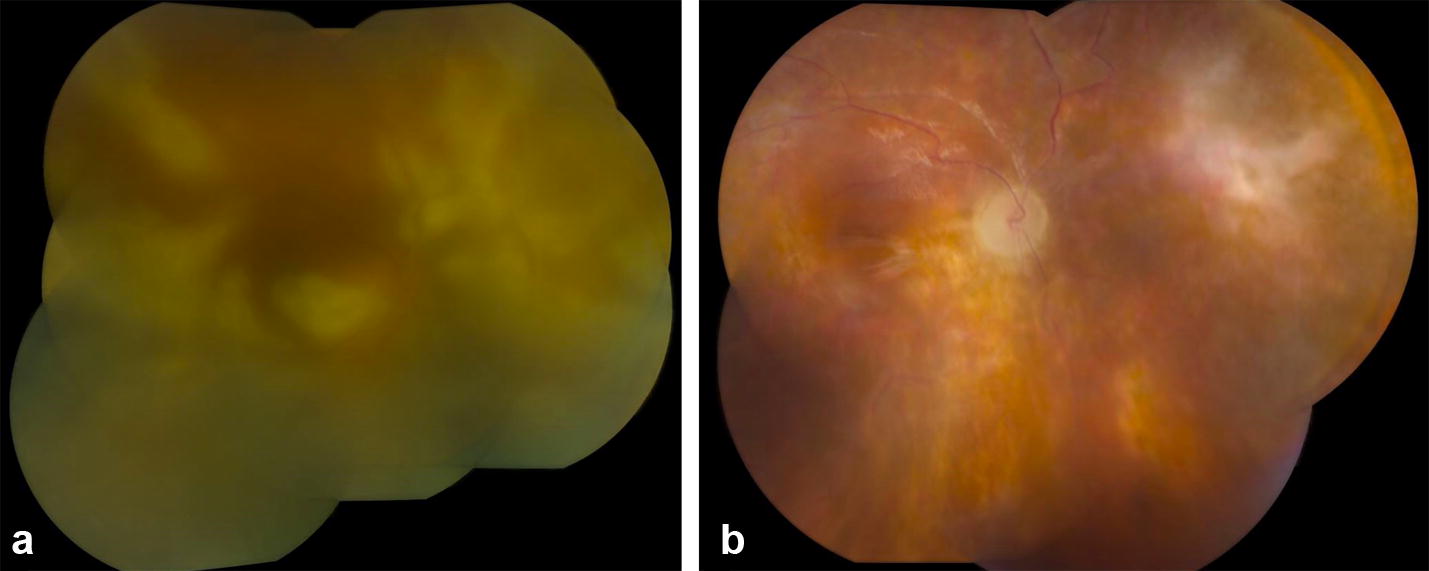
Color fundus photo montage of the right eye of patient three demonstrates a hazy view secondary to vitritis and diffuse, fulminant retinal necrosis (**a**). Following anti-toxoplasmosis therapy and retinal detachment repair, the retina is attached although there is severe vascular attenuation, optic nerve pallor, and fibrosis with chorioretinal scarring superonasally (**b**)

The patient’s presenting visual acuities were 20/600 in the right eye and 20/15 in the left eye. A relative afferent pupillary defect was present in the right eye. Slit lamp exam demonstrated 2+ anterior chamber and 1+ vitreous cell in the right eye. Dilated examination of the right eye showed 2+ vitreous haze and diffuse retinal whitening. The left eye was normal. Intravitreal foscarnet was administered, and an aqueous sample was obtained for PCR for HSV, VZV, CMV, and toxoplasmosis. Prednisone was tapered over to 10 mg/day over a 1 week period. Serologic testing showed toxoplasmosis IgG of 21.2 IU/mL and Toxoplasmosis IgM positive at 1.2 antibody titer (Normal reference < 0.9). An FTA-ABS was negative. One week later, PCR testing of the aqueous humor was negative for viruses and positive for toxoplasmosis DNA. While awaiting PCR testing results, the retinitis had continued to worsen with intravitreal foscarnet and ganciclovir injections (Fig. 3). Trimethoprim/sulfamethoxazole therapy was initiated, and clindamycin was injected intravitreally; however, the patient developed a combined tractional and rhegmatogenous retinal detachment, which was repaired with vitrectomy, endolaser, membrane peel, and silicone oil instillation. Postoperatively, she was treated with pyrimethamine, clindamycin, and folinic acid. Clindamycin was elected at this point in the patient’s treatment instead of trimethoprim/sulfamethoxazole because of concerns that the patient was developing chronic kidney disease with an elevation of creatinine to 1.2 mg/dL and glomerular filtration rate of 45 mL/min/1.73 m^2^. The patient’s retinitis remained inactive, but her vision remained hand motions at 18-month follow-up (Fig. 3).

## Discussion

Ocular toxoplasmosis in immunocompromised patients may present with greater severity and a diffuse, necrotizing appearance that differs from the typical “headlight in a fog” appearance. Local corticosteroids may reduce the ocular immune response and lead to the rapid evolution of ocular toxoplasmosis to a fulminant retinal necrosis [3]. These patients represent a diagnostic challenge, and the differential diagnosis should include herpetic retinitis, cytomegalovirus retinitis, syphilis, bacterial or fungal endophthalmitis, as well as toxoplasmosis. Because of the broad differential diagnosis and the potential for vision loss, prompt evaluation with aqueous PCR for CMV, HSV, VZV, and toxoplasmosis along with early initiation of empiric therapy are essential [4]. Ocular fluid may be analyzed with immunoglobulin assays, but PCR may be more sensitive for pathogen diagnosis, particularly in immunocompromised patients who may have a reduced host antibody response.

In our series, PCR was diagnostic in two of three cases presented, one from the aqueous fluid (patient three) and one from a vitreous sample (patient two). Prior studies of aqueous PCR for toxoplasmosis have reported a range of positivity from 18 to 67% [2, 5]. The sensitivity of PCR testing for toxoplasmosis may be greater with vitreous samples compared to aqueous humor.

Interestingly, our series of patients showed a range of toxoplasmosis serologic testing results, which may confound the diagnosis in some cases. Patient 1 showed no serologic evidence of Toxoplasmosis IgG or IgM and was only diagnosed via histopathologic evaluation of vitreous fluid. Given her negative toxoplasmosis PCR from the aqueous humor, a high index of suspicion and detailed ophthalmic pathologic evaluation were eventually needed to identify toxoplasmosis bradyzoites. Patient 3 showed an extremely high IgG level, as well as a positive IgM response. While it is unclear whether this represents a true, acute infection, the antibody response was reflective of a robust systemic antibody response targeting high levels of toxoplasmosis antigen, despite the patient receiving multiple immunosuppressive medications.

Besides serology and PCR testing, histopathologic assessment of vitreous samples may also be extremely helpful to establish a diagnosis of toxoplasmosis in select cases. Patient 1 demonstrated progressive chorioretinitis, which was suspicious for toxoplasmosis and a diagnostic vitrectomy was elected. Clear communication with the ophthalmic pathologist is essential in these scenarios to detect the pathogen and initiate appropriate therapy.

## Conclusion

In summary, we recommend consideration of toxoplasmosis in the differential of necrotizing retinitis in immunocompromised patients and elderly patients, particularly if local corticosteroids have recently been administered. A systematic approach that includes aqueous or vitreous PCR and potentially diagnostic vitrectomy for histopathologic evaluation may be beneficial to establish a diagnosis in these challenging cases.

## Data Availability

Not applicable.
